# Polysarcosine-Based Lipids: From Lipopolypeptoid Micelles to Stealth-Like Lipids in Langmuir Blodgett Monolayers

**DOI:** 10.3390/polym8120427

**Published:** 2016-12-09

**Authors:** Benjamin Weber, Christine Seidl, David Schwiertz, Martin Scherer, Stefan Bleher, Regine Süss, Matthias Barz

**Affiliations:** 1 Institute of Organic Chemistry, Johannes Gutenberg University of Mainz, Duesbergweg 10-14, 55128 Mainz, Germany; b.weber@uni-mainz.de (B.W.); cseidl@students.uni-mainz.de (C.S.); daschwie@students.uni-mainz.de (D.S.); m.scherer@uni-mainz.de (M.S.); 2 Department of Pharmaceutical Technology and Biopharmacy, Institute of Pharmaceutical Sciences, Albert Ludwigs University of Freiburg, Sonnenstraße 5, 79104 Freiburg im Breisgau, Germany; stefan.bleher@pharmazie.uni-freiburg.de (S.B.); regine.suess@pharmazie.uni-freiburg.de (R.S.)

**Keywords:** polysarcosine, polypeptoids, surfactants, lipids, NCA polymerization, PSarcosinylated lipids

## Abstract

Amphiphiles and, in particular, PEGylated lipids or alkyl ethers represent an important class of non-ionic surfactants and have become key ingredients for long-circulating (“stealth”) liposomes. While poly-(ethylene glycol) (PEG) can be considered the gold standard for stealth-like materials, it is known to be neither a bio-based nor biodegradable material. In contrast to PEG, polysarcosine (PSar) is based on the endogenous amino acid sarcosine (*N*-methylated glycine), but has also demonstrated stealth-like properties in vitro, as well as in vivo. In this respect, we report on the synthesis and characterization of polysarcosine based lipids with C_14_ and C_18_ hydrocarbon chains and their end group functionalization. Size exclusion chromatography (SEC) and matrix-assisted laser desorption/ionization time-of-flight mass spectrometry (MALDI-TOF MS) analysis reveals that lipopeptoids with a degree of polymerization between 10 and 100, dispersity indices around 1.1, and the absence of detectable side products are directly accessible by nucleophilic ring opening polymerization (ROP). The values for the critical micelle concentration for these lipopolymers are between 27 and 1181 mg/L for the ones with C_18_ hydrocarbon chain or even higher for the C_14_ counterparts. The lipopolypeptoid based micelles have hydrodynamic diameters between 10 and 25 nm, in which the size scales with the length of the PSar block. In addition, C_18_PSar_50_ can be incorporated in 1,2-distearoyl-*sn*-glycero-3-phosphocholine (DSPC) monolayers up to a polymer content of 3%. Cyclic compression and expansion of the monolayer showed no significant loss of polymer, indicating a stable monolayer. Therefore, lipopolypeptoids can not only be synthesized under living conditions, but my also provide a platform to substitute PEG-based lipopolymers as excipients and/or in lipid formulations.

## 1. Introduction

Amphiphilic molecules and polymers are commonly applied to lower the surface tension (or interfacial tension) between two liquids or between a liquid and a solid, which enables their use as detergents, wetting agents, emulsifiers, foaming agents, and dispersants [1,2]. From a structural point of view, these polymers can be divided into two classes, which are characterized by the relative distribution of hydrophilic and lipophilic units. Macromolecules based on intrinsically amphiphilic repeating units are summarized as “polysoaps”, whereas polymers with strictly separated parts are called “macrosurfactants” [3,4,5,6]. These macrosurfactants are commonly amphiphilic block copolymers or lipopolymers, in which a hydrocarbon chain of 12–18 units is attached to a hydrophilic polymer. With respect to sustainability, bio-based amphiphiles, such as lipopeptides, have gained pronounced attention as they are based on renewable raw materials [7]. This enormous potential was already recognized half a century ago [8,9,10] and, thus, several amino acid or peptide based amphiphiles have been investigated, in which fatty acid chains, as well as amino acids or peptides, can vary in composition and length [11]. 

In addition, PEGylated lipids or alkyl ethers represent an important class of non-ionic lipopolymers and are key ingredients for the preparation of long-circulating liposomes, since a significant step in the development of long-circulating liposomes came with the incorporation of the synthetic polymer poly-(ethylene glycol) (PEG) in liposome compositions. The presence of PEG on the surface of the liposomal carriers has been shown to extend blood-circulation time while reducing mononuclear phagocyte system uptake (stealth liposomes). Despite the enormous achievements of PEGylated lipids, several groups have reported immune responses towards PEG and PEGylated lipids, leading to the accelerated blood clearance (ABC) phenomenon [12,13,14]. Moreover, PEG is not degradable in vivo and relies on complete excretion to avoid storage diseases [15]. Consequently, the finding of alternatives to PEG is a growing field of research [16]. Among various PEG surrogates, polypeptides and polypeptoids are attracting more and more attention [16,17,18]. The polypeptoid polysarcosine (PSar) seems to be particularly interesting because it is, on one hand, based on the endogenous *N*-substituted amino acid, sarcosine (*N*-methylated glycine), and on the other hand, sarcosine can be easily synthesized by a simple nucleophilic substitution reaction of bromo- or chloroacetic acid and methylamine [19]. Furthermore, PSar can be synthesized under living conditions from the corresponding α-amino acid *N*-carboxy anhydride (NCA) [19,20,21] and has already demonstrated possessing stealth-like properties comparable to PEG [18,22,23,24,25,26]. Interestingly, lipopeptides, as non-ionic and bio-based systems, have been practically overlooked. So far only Gallot and coworkers have reported on the synthesis of PSar-based lipopolymers (lipopeptoids). In 1986 they reported the synthesis of lipopeptoids using aliphatic amines to initiate the ring opening polymerization (ROP) of the Sar NCA in chloroform [27,28]. Surprisingly, they had to fractionate the final lipopolypeptoid yielding different fractions with degrees of polymerization from 10 to 60. Due to the living nature of Sar NCA ROP, one would expect that such degrees of polymerization can be directly obtained by adjusting the monomer to initiator ratio. To validate our expectation, we carried out the synthesis of lipopolypeptoids based on either tetradecylamine (C_14_) or stearylamine (C_18_).

In this work, we report the synthesis and end group functionalization of PSar based lipopolypeptoids with C_14_ and C_18_ hydrocarbon tails. The lipopolypeptoids have a PSar block with chain lengths (*X*_n_) from 10–100. Furthermore, we introduce a synthetic pathway, which allows polymerization of such systems on 50–100 g scale. The final lipopolymers are characterized by ^1^H NMR, ^1^H-DOSY NMR, SEC and MALDI-TOF mass spectrometry to ensure the living nature of the ring opening polymerization. We also report on the critical micelle concentration (CMC) of the synthesized systems, characterize the aggregates by dynamic light scattering, investigate cellular toxicities, and report on the incorporation of 1, 2, and 3 mol % of PSar45-stearylamine into Langmuir-Blodgett monolayers of 1,2-distearoyl-*sn*-glycero-3-phosphocholine (DSPC). 

## 2. Materials and Methods

*n*-Hexane was distilled from Na/K and ethyl acetate from CaH_2_. Dimethylformamide (DMF) was purchased from Acros Organics (Geel, Belgium) and dried over BaO and molecular sieves (3 Å), fractionally distilled under vacuum at 40 °C and stored at −80 °C under the exclusion of light. Prior to use, DMF was degassed in vacuum to remove traces of dimethylamine. Hexafluoroisopropanol (HFIP) was purchased from Fluorochem (Hadfield Derbyshire, UK). Millipore water was prepared by a MILLI-Q^®^ Reference A^+^ System (Darmstadt, Germany). Octadecylamine and was purchased from Fluka (St. Gallen, Switzerland) and was dried at 40 °C under vacuum (1 × 10^−3^ mbar) for 24 h. Diphosgene was purchased from Alfa Aesar (Ward Hill, MA, USA) and deuterated solvents from Deutero GmbH (Kastellaun, Germany). Other chemicals were purchased from Sigma-Aldrich (Taufkirchen, Germany) and used as received unless otherwise stated. 1,2-distearoyl-*sn*-glycero-3-phosphocholine (DSPC) was purchased from Avanti Polar Lipids (Alabaster, Al, USA) and used without purification. Roswell Park Memorial Institute (RPMI) cell medium and FCS was purchased from Merck Millipore (Darmstadt, Germany). HeLa cells were obtained from DSMZ (German Collection of Microorganisms and Cell Cultures, Braunschweig, Germany).

^1^H NMR spectra were recorded on a Bruker (Billerica, MA, USA) AC 400 at a frequency of 400 MHz respectively. Two-dimensional NMR spectra as ^1^H DOSY were recorded on a Bruker Avance III HD 400 at 400 MHz. All spectra were recorded at room temperature (25 °C) and calibrated using the solvent signals. Melting points were measured using a Mettler FP62 melting point apparatus at a heating rate of 2.5 °C·min^−1^. Gel permeation chromatography (GPC) was performed with hexafluoroisopropanol (HFIP) containing 3 g·L^−1^ potassium trifluoroacetate (KTFA) as the eluent at 40 °C and a flow rate of 0.8 mL·min^−1^. The columns were packed with modified silica (PFG column particle size: 7 μm, porosity: 100 and 1000 Å). Polymethylmethacrylate (PMMA) standards (Polymer Standards Services GmbH (Mainz, Germany)) were used for calibration and toluene was used as the internal standard. A refractive index detector (G1362A RID) and an UV-VIS detector (at 230 nm unless otherwise stated; Jasco (Gross-Umstadt, Germany) UV-2075 Plus) were used for polymer detection. MALDI-TOF mass spectra [29] were recorded using a Bruker Reflex II MALDI-TOF mass spectrometer equipped with a 337 nm N_2_ laser. Acceleration of the ions was performed with pulsed ion extraction (PIE, Bruker) at a voltage of 20 kV. The analyzer was operated in reflection mode and the ions were detected using a microchannel plate detector. Mass spectra were processed by the X-TOF 5.1.0 software (Bruker (Billerica, MA, USA)). A solvent-free sample preparation was performed using trans-2-[3-(4-*tert*-Butylphenyl)-2-methyl-2-propenylidene]malononitrile (DCTB) as the matrix and sodium trifluoroacetate as the cationizing salt. Calibration was carried out using a C_60_/C_70_ fullerene mixture. Infrared (IR) spectroscopy was performed on a Jasco FT/IR-4100 with an ATR sampling accessory (MIRacle, Pike Technologies, Madison, WI, USA) and Spectra Manager 2.0 (Jasco, Gross-Umstadt, Germany) was used for integration.

Surface pressure-area (π vs. *A*) isotherms were obtained using a Nima Langmuir-Blodgett trough (KSV Nima, (Espoo, Finland), Coventry, type 611) secured inside an acrylic glass box (Bayer, Leverkusen, Germany) as a dust shield. The total trough surface area was 200 mm × 100 mm, and the total trough volume was approximately 150 mL. The effective trough area was controlled by two hydrophobic barriers that compressed the spread film symmetrically and bilaterally at a rate of 5 cm^2^/min. Millipore water was used as subphase in all trials. For all experiments, the subphase temperature was 25 ± 0.1 °C (15-min delay after the water was filled in and the lipid solution was spread). Prior to each trial, the water surface was cleaned by aspirating off any residue, such that the measured surface pressure remained <0.1 mN/m over a full compression. The Langmuir-Blodgett (LB) components were cleaned with absolute ethanol and chloroform before each experiment, and the deionized water subphase was replaced after each measurement. Surface pressure measurements were taken from a Wilhelmy plate (perimeter of 20 mm × 10 mm) made out of chromatography paper, which was washed several times with absolute chloroform prior to each trial to ensure cleanliness. Dynamic light scattering measurements were performed at 25 °C using a Malvern (Malvern, UK) Zetasizer NanoZS with a He/Ne laser (633 nm) at a fixed angle of 173°.

Synthesis of sarcosine *N*-carboxyanhydride. The synthesis of sarcosine NCA was adapted from literature and modified. A total of 14.92 g (167.4 mmol) sarcosine, dried under vacuum for 1 h, was weighed into a pre-dried, three-neck, round-bottom flask. A total of 300 mL of absolute tetrahydrofurane (THF) was added under a steady flow of nitrogen, 16.2 mL (134 mmol) of diphosgene was added slowly via syringe, and the nitrogen stream was reduced. The colorless suspension was mildly refluxed for 3 h, yielding a clear solution. Afterward, a steady flow of dry nitrogen was led through the solution for another 3 h while the outlet was connected to two gas washing bottles filled with aqueous NaOH solution to neutralize phosgene. The solvent was evaporated under reduced pressure, yielding a brownish oil as a crude reaction product. The oil was dried under reduced pressure (1 × 10^−3^ mbar for 2 h) to obtain an amorphous solid, free of phosgene and HCl, confirmed by testing against a silver nitrate solution. The crude product was redissolved in 40 mL of THF and precipitated with 300 mL of dry *n*-hexane. The solution was cooled to −18 °C and stored for 18 h to complete precipitation. The solid was filtered under dry nitrogen atmosphere and dried in a stream of dry nitrogen for 60–90 min and afterwards under high vacuum for 2 h in the sublimation apparatus. The crude product was sublimated at 85 °C and 1 × 10^−3^ mbar. The product was collected from the sublimation apparatus in a glovebox on the same day. The purified product (110 mmol, 65% yield, colorless crystallites; melting point: 102–104 °C (lit: 102–105 °C)) was stored in a Schlenk tube at −80 °C and only handled in a glovebox. 

^1^H NMR (300 MHz, CDCl_3_): δ/ppm = 4.22 (2H, s, –CH_2_–CO–), 2.86 (3H, s, –CH_3_).

Synthesis of polysarcosine. Under nitrogen counter flow, Sar-NCA was transferred into a pre-dried Schlenk tube equipped with a stir bar and again dried under high vacuum for 1 h. Then, the NCA was dissolved in dry DMF to yield a solution of 100 mg/mL with respect to the NCA. 1/*n* equivalent of either tetradecyl amine or stearyl amine was dissolved in pre-dried THF and added to the NCA solution. The solution was stirred at room temperature and kept at a constant pressure of 1.25 bar of dry nitrogen via the Schlenk line to prevent impurities from entering the reaction vessel while allowing CO_2_ to escape. Completion of the reaction was confirmed by Fourier transform infrared (FTIR) spectroscopy (disappearance of the NCA peaks (1853 and 1786 cm^−1^)). After completion of the reaction, the polymer was precipitated with cold ether and centrifuged (4500 rpm at 4 °C for 15 min). After discarding the liquid fraction, new ether was added and the polymer was resuspended in a sonic bath. The suspension was centrifuged again and the procedure was repeated. After complete DMF removal by the resuspension steps, the polymer was dissolved in water and lyophilized, obtaining a colorless, stiff and porous solid. 

^1^H NMR (400 MHz; DMSO-*d*_6_): δ/ppm: 4.43–3.83 (14H; br; (2*n*)–CO–CH_2_–NH–); 3.14–2.65 (23H; br; (3*n*)–N–CH_3_–); 1.53–1.12 (32H; br; –CH_2_–(CH_2_)_16_–CH_3_); 0.86 (3H; t; –CH_2_–CH_3_).

Synthesis of carboxy functionalized polymers. The polymer was dissolved in dry DMF with 10 eq. (with respect to the polymer end group) of diisopropylethylamine (DIPEA) and stirred for 30 min. To this solution the 5 eq. succinic acid anhydride was added and stirred overnight at room temperature. The excess of DIPEA and succinic anhydride were removed by dialysis and the product was lyophilized. Complete removal was verified by DOSY ^1^H NMR.

^1^H NMR (400 MHz; DMSO-*d*_6_): δ/ppm: 4.69–3.72 (97H; br; (2*n*)–CO–CH_2_–NH–); 3.10–2.66 (152H; br; (3*n*)–N–CH_3_–); 2.42–2.25 (4H; br; –CO–(CH_2_)_2_–COOH); 1.47–1.17 (32H; br; –CH_2_–(CH_2_)_16_–CH_3_); 0.86 (3H; t; –CH_2_–CH_3_).

Synthesis of acetylated polymers. The polymer was dissolved in dry DMF with 10 eq. of DIPEA and stirred for 30 min. To this solution the 5 eq. acetic anhydride or the FITC was added and stirred overnight at room temperature. Excess DIPEA and acetic acid anhydride were removed by dialysis and the product was lyophilized. Complete removal was verified by ^1^H-DOSY NMR.

^1^H NMR (400 MHz; DMSO-*d*_6_): δ/ppm: 4.55–3.77 (99H; br; (2*n*)–CO–CH_2_–NH–); 3.22–2.63 (154H; br; (3*n*)–N–CH_3_–); 2.06–1.90 (3H; br; –NCH_3_–CO–CH_3_); 1.49–1.14 (32H; br; –CH_2_–(CH_2_)_16_–CH_3_); 0.86 (3H; t; –CH_2_–CH_3_).

Synthesis of FITC labeled polymers. The polymer was dissolved in dry DMF with 10 eq. of DIPEA and stirred for 30 min. To this solution 2 eq. FITC was added and stirred overnight at room temperature. Excess DIPEA and FITC were removed by dialysis and the product was lyophilized. Complete removal was verified by ^1^H-DOSY NMR.

^1^H NMR (400 MHz; DMSO-*d*_6_): δ/ppm: 10.32–9.87 (1H; br; FITC–COOH); 8.37–7.37 (3H; br; aromatic –CH–C–COOH–; aromatic CH–CH–COH; aromatic CH–CH–CNHR); 6.74–6.24 (6H; br; –CH–CO–CH–CH–; –COH–CH–COR–; CH–CH–CNHR); 4.81–3.71 (93H; br; (2*n*)–CO–CH_2_–NH–); 3.14–2.65 (142H; br; (3*n*)–N–CH_3_–); 1.54–1.10 (32H; br; –CH_2_–(CH_2_)_16_–CH_3_); 0.86 (3H; t; –CH_2_–CH_3_).

CMC measurements. CMCs have been determined with a Dataphysics (Filderstadt, Germany) ring tensiometer (DCATIIEC) at 25 °C. It was calibrated against deionized water purified with a Milli-Q system (Merck Millipore, Darmstadt, Germany) to 18.2-MΩ cm resistivity and TOC <5 ppb. All samples were aged for 30 min prior to use.

Dynamic light scattering measurements: Lipopeptoids were dissolved in phosphate buffered saline (PBS) to yield 0.1 mg/mL. Prior to measurement samples were aged for 30 min. 

Cellular Toxicity: Toxicity studies were carried out using the CellTiter-Glo^®^ Luminescent Cell Viability Assay by Promega. The assay was carried out following the manufacturers’ protocol. HeLa cells were cultured in RPMI medium with 10% heat-inactivated fetal bovine serum (FCS). Cells were harvested at 60%–70% confluence, incubated at 37 °C, 95% relative humidity (rh) and 5% CO_2_ for 24 h in a 24-well plate with lipopeptoids. Lipopeptoids were dissolved in PBS. Medium was replaced 1 h prior to experiments. The experiments were performed in triplicate. Data was normalized to the untreated control.

Langmuir Blodgett layer formation: Solutions of the polymer-lipid mixtures in chloroform were spread on the subphase by using a microsyringe (Kloehn, Las Vegas, NV, USA). In a typical experiment, 20–30 μL of the solution was spread dropwise onto the water surface so that a constant mass of lipid was deposited for each trial. The spreading solution was deposited at regularly spaced locations on the trough. In all trials, a 15-min evaporation period between the last deposited drop of solution and the beginning of compression was employed to ensure complete solvent evaporation.

## 3. Results

### 3.1. Synthesis of Lipopolypeptoids

**Figure 1:**
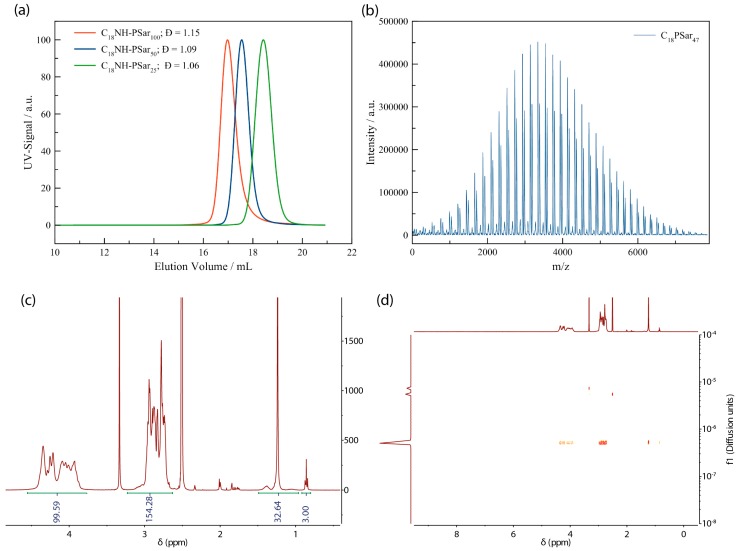
Exemplary polymer analysis of lipopolypeptoids (**a**) HFIP SEC of C_18_PSar_34_, C_18_PSar_45_, and C_18_PSar_117_; (**b**) MALDI TOF MS of C_18_PSar_47_; (**c**) ^1^H NMR of C_18_PSar_47_; and (**d**) ^1^H DOSY NMR of C_18_PSar_47_.

The synthesis of 100 mg to 1 g of lipopolymer was conducted using freshly sublimated Sar-NCA, purified solvent (DMF) and initiator (tetradecyl or stearyl amine). The polymerizations were carried out at room temperature with a monomer concentration of 0.1 g/mL. After complete monomer conversion was ensured by FTIR-measurements (disappearance of NCA attributed carbonyl vibration band at 1786 and 1850 cm^−1^) lipopeptoids were precipitated in cold diethylether. Afterwards, lipopolymers were dried by lyophilization from water and analyzed by ^1^H NMR, HFIP SEC, MALDI-TOF, and ^1^H-DOSY NMR. ^1^H NMR experiments displayed that the deviation of the obtained degrees to those calculated are below 10% (Table 1). In hexafluoroisopropanole (HFIP) SEC the synthesized lipopolymers indicate a symmetric narrowly-distributed molecular weight distribution. The PMMA equivalent molecular weights are in the range of 5 to 25 kg/mol, while dispersities are between 1.05 and 1.13 (see Table 1). Furthermore, SEC clearly demonstrates that the hydrodynamic volume scales with the degree of polymerization (Figure 1a). An influence of the initiator on the control over polymerization was not detectable, as both aliphatic amines lead to a well-controlled polymerization. To ensure the formation of lipopolymers and the absence of PSar homopolymers ^1^H-DOSY NMR experiments have been carried out. The diffusion ordered NMRs confirm the absence of low molecular weight or high molecular weight side-products, since only a single diffusing polymer species is detected, which contains all PSar, as well as lipid attributed proton signals. 

**Scheme 1:**
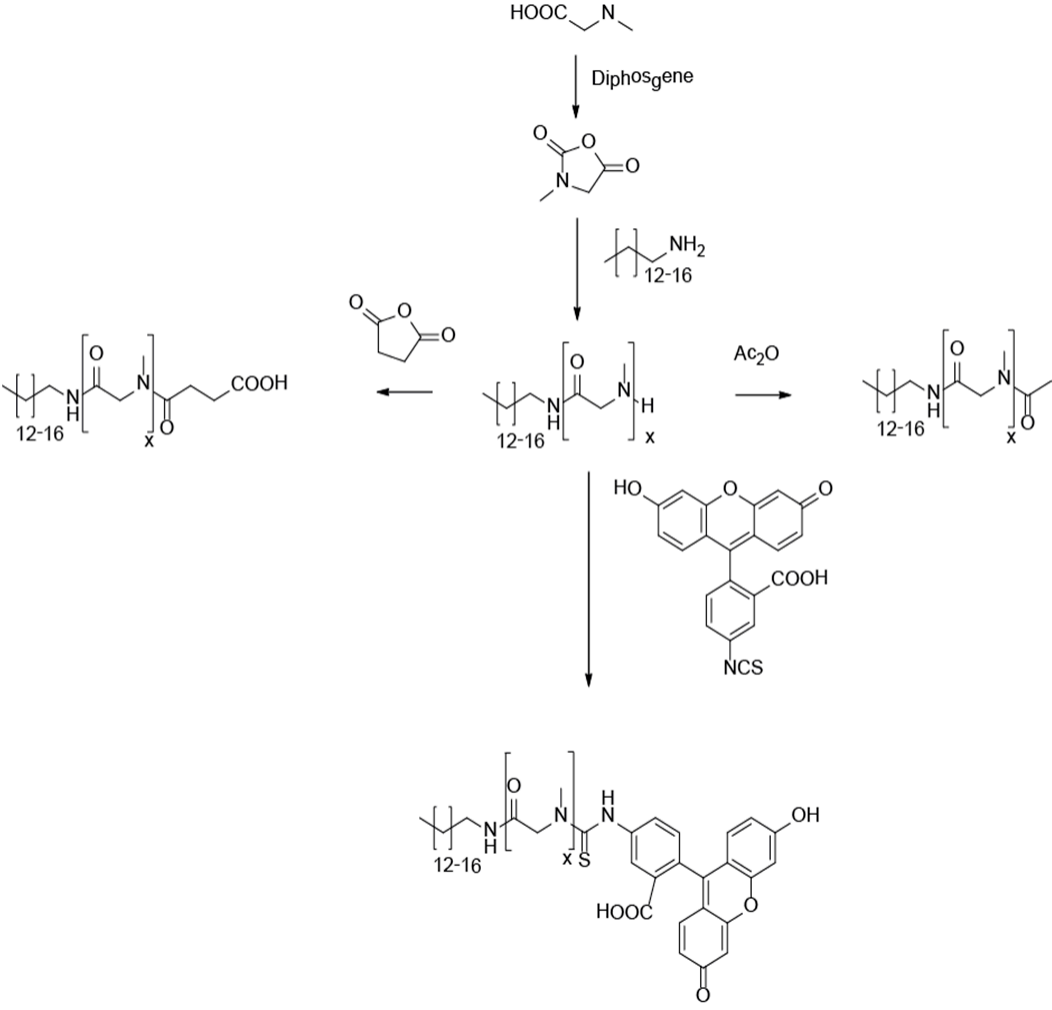
A synthetic pathway to heterotelechelic lipopolypeptoids.

**Figure A1:**
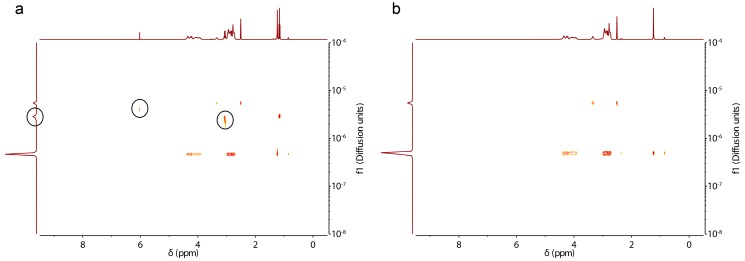
DOSY ^1^H NMR of C_18_PSar_45_COOH (**a**) before, and (**b**) after removal of succinic anhydride.

In the next step, the methylamine end groups were quenched with fluoresceine isothiocyanate (FITC), succinic acid anhydride (COOH functionality), or acetic acid anhydride (neutral end group) in the presence of diisopropylethylamine (DIPEA) (Scheme 1). While acetylated PSar could be used as a stealth-only material, carboxylic acid-functionalized lipopolypeptoids are accessible to further modifications since targeting moieties e.g., antibodies or sugars can be attached. Labeling with a dye, in this case FITC, will allow analysis of these formulations with fluorescent techniques, e.g., fluorescent correlation spectroscopy, confocal microscopy, and fluorescence activated cell sorting. To monitor the end group modification efficiency further ^1^H-DOSY NMR experiments have been conducted. This method cannot only help to ensure that end group modification is complete, but also confirm the successful removal of the small molecules used for the polymer modification during workup (dialysis). (Appendix A Figure A1) With respect to the limits of ^1^H NMR spectroscopy, these experiments reveal the absence of impurities as well as the quantitative conversion of PSar end groups. 

After the synthesis, solution properties of lipopolypeptoids are investigated, which are the critical micelle concentration (CMC) and the hydrodynamic diameter (*D*_h_) in aqueous solution by dynamic light scattering (DLS). First, we calculated the hydrophilic-lipophilic balance (HLB) for the synthesized lipopolymers.

### 3.2. Characterization, Solution Properties, and Cellular Toxicity of Lipopolypeptoids

According to W.C. Griffin the hydrophilic-lipophilic balance (HLB) is defined as [30]:

(1) $$\text{HLB}=20\times (1-\frac{M_{lipohilic}}{M_{lipopeptoid}})$$

Therefore, the HLB values are between 15 and 20 for the synthesized lipopolymers, which is a HLB range for solubilizing agents in general (see Table 2). Solubilizing agents are a class of amphiphiles, which are hardly able to incorporate water soluble substances into micellar structures. This process is called micellar solubilization, or, briefly, solubilization [31]. In comparison to other amphiphiles, solubilizing agents have higher HLB values and biocompatible ones are often used to solubilize hydrophobic drugs [32].

**Figure 2:**
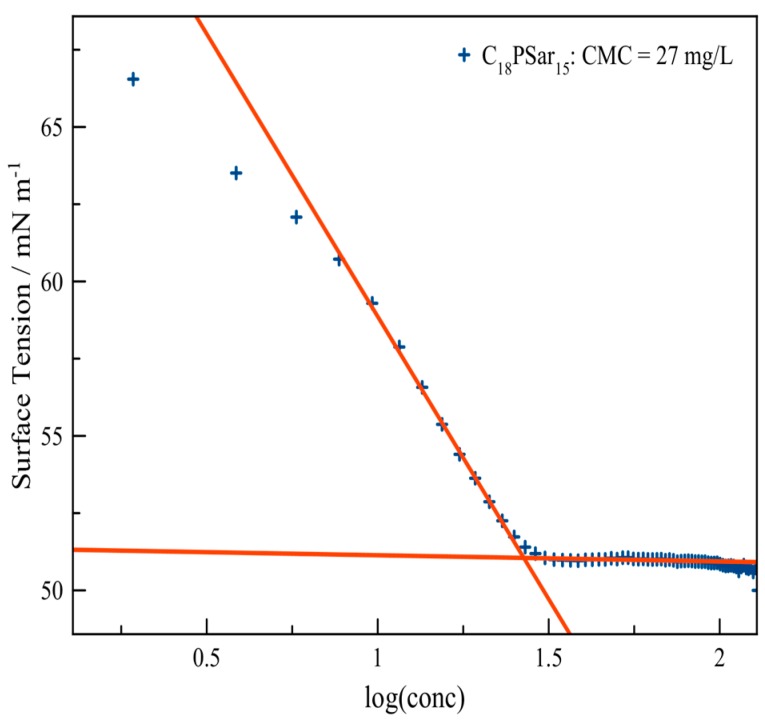
Exemplary CMC measurement shown for C_18_PSar_15_. CMC was determined by ring tensiometry. Intersection of the two lines represents the CMC value.

With respect to their HLB values, all synthesized lipopolymers can be considered as solubilizing agents, which raises the question of PSar chain length dependency of CMCs and hydrodynamic diameter of micelles. Therefore, ring tensiometry was used to determine CMC values of the synthesized lipopolypeptoids. In the applied tensiometer setup the surface tension at about 70 different concentrations was measured. With increasing amounts of surfactant the surface tension of the solution decreases. Once the CMC is reached the surface tension remains constant and micelles start to form. All additional surfactants added to the system increase the micellar fraction (Figure 2). Within the series of C_18_ lipopolypeptoids, the CMC increases with the degree of polymerization of the PSar block and, thus, with HLB values from 0.027 (C_18_PSar_12_) to 1.181 g/L (C_18_PSar_117_) (See Table 1). As expected, lipopolypeptoids with C_14_ alkyl chain have significantly higher CMCs than those with C_18_ alkyl chain. The aggregation concentration is so high that only for C_14_PSar_11_ a CMC could be determined in the applied concentration range. Thus, CMCs of the other C_14_ lipopolymers are above 1.500 g/L. 

**Figure 3:**
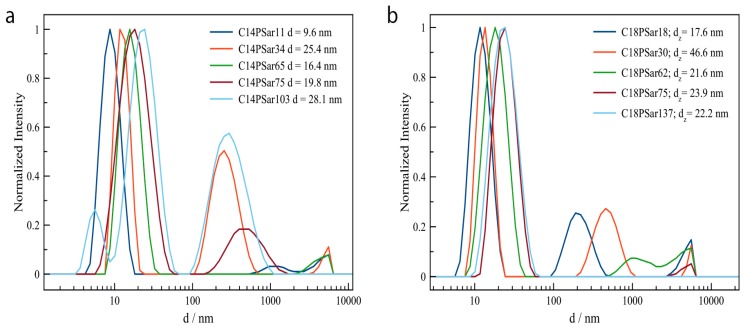
Intensity-weighted diameter distribution of lipopolypeptoids in aqueous solution determined by dynamic light scattering (DLS). (**a**) Series of tetradecyl amine (C_14_)-based lipopolypeptoids; and (**b**) series of stearyl amine (C_18_)-based lipopolypeptoids.

After determining CMC values, the synthesized lipopolypeptoids micelles have been further investigated. Therefore, a concentration of 1 g/L was chosen, which is well above the CMC of the corresponding C_18_ lipopolymers. Only the lipopolymers with a C_14_ alkyl chain and the C_18_PSar_117_ have been characterized at a concentration of 10 g/L. The series of C_18_ lipopolypeptoids formed aggregates, which increase in size with the degree of polymerization. The aggregates have diameters between 10.1 nm (C_18_PSar_12_) and 24.9 nm (C_18_PSar_117_) (see Table 2). For the C_14_ lipopolypeptoids the trend is also confirmed, since those micelles have diameters between 9.6 nm (C_14_PSar_11_) and 28.1 nm (C_14_PSar_103_). C_14_PSar_103_ also shows a unimer fraction next to the micellar fractions (Figure 3). Micelles formed by C_18_ and C_14_ lipopolypeptoids have a comparable diameter at comparable degrees of polymerization. For the PSars with *X*_n_ < 35 a fraction of several hundred nm is observed. The number-weighted distribution does not display these fractions (Figure A3), which is due to the overestimation of large fractions in intensity weighted plotting. Since these polymers have only a single alkyl chain, hydrophobic stabilization of the micelles is very low. This leads to relatively high CMCs and, consequently, to a dynamic system with high exchange rates, which seems to be the reason for the formation of more complex aggregates.

**Figure 4:**
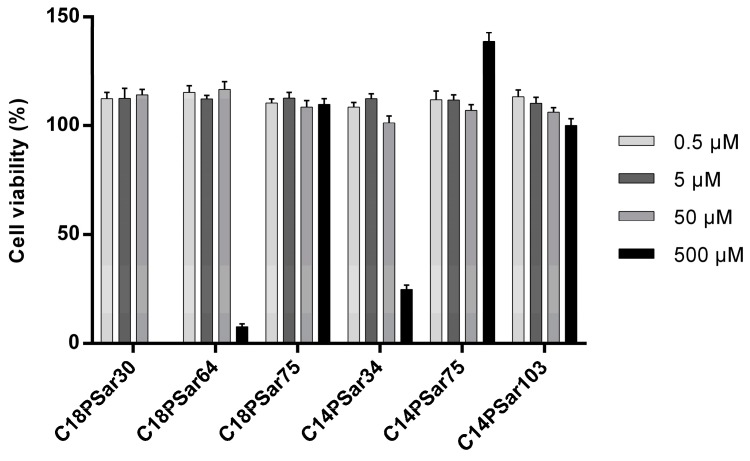
CellTiter-Glo^®^ (CTG) assay toxicity studies of unformulated amphiphilic PSars in HeLa cells in the concentration range from 0.5 up to 500 μM. Data was normalized to the untreated control. For better comparability molar concentrations were chosen. 1.2 mg/mL (C_18_PSar_30_) and 4.2 mg/mL (C_14_PSar_103_) correspond to 500 μmol.

In the next step and with a view using lipopolypeptoids as excipients in drug formulations, the cellular toxicity of lipopolypeptoids was investigated in HeLa cells using the CellTiter-Glo^®^ assay. This assay is a method of determining the number of viable cells based on quantitation of the ATP values present, an indicator of metabolically active cells. The quantification relies on a proprietary thermostable luciferase, which generates a stable “glow-type” luminescent signal depending on ATP levels. In relation to untreated cells as the positive control, relative cell viability can be determined. In this case lipopolypeptoids showed no toxicity up to a concentration of 50 μM. For a concentration of 500 μM (1.2 to 4.2 mg) the lipopolymers with low degrees of polymerization showed toxicity, which was not observed for lipopolymers with high PSar content (Figure 4). 

### 3.3. Formation and Characterization of Lipopolypeptoid Containing Langmuir-Blodgett Monolayers

In the last part of this study, and with regard to the incorporation of lipopolypeptoids in liposomes to provide stealth-like properties, it was investigated to which extent lipopolypeptoids can be incorporated into lipid membranes of 1,2-distearoyl-*sn*-glycero-3-phosphocholine (DSPC). On a Langmuir-Blodgett trough solutions of the lipopolymer-lipid mixture in chloroform were spread on the subphase. After evaporation of the chloroform, the area was compressed with a speed of 5 cm^2^/min. Plotting the surface pressure over the area, a similar behavior is observed for a polymer content up to 3 mol %. When the compression starts pure DSPC lipids are in the gas analogous phase. At a pressure from 0 to 3.8 mN/m lipids are in the liquid analogous phase having an average area of 70 Å^2^/mol. At 55 Å^2^/mol the slope of the curve rises and the solid analogous phase is reached. For the mixture bearing 1% lipopolypeptoids the phase transitions remain at the same area/molecule, but the transition from liquid to solid analogous phase takes place at 6.7 mN/m. Having a mixture with 2% lipopolypeptoids the onset point of the liquid analogous phase starts at 92 Å^2^/mol. The surface pressure rises slowly to 8.15 mN/m and a small plateau is reached at 59 Å^2^/mol (coexistence of liquid and solid analogous phase). Transition to solid analogous phase remains at 55 Å^2^/mol at a surface pressure of 10.7 mN/m. The phase transition to liquid analogous phase in the mixture with 3% C_18_PSar_47_ starts at 100 Å^2^/mol. The coexistence of solid and liquid analogous phase end at a surface pressure of 7.8 mN/m and 60 Å^2^/mol. 

**Figure 5:**
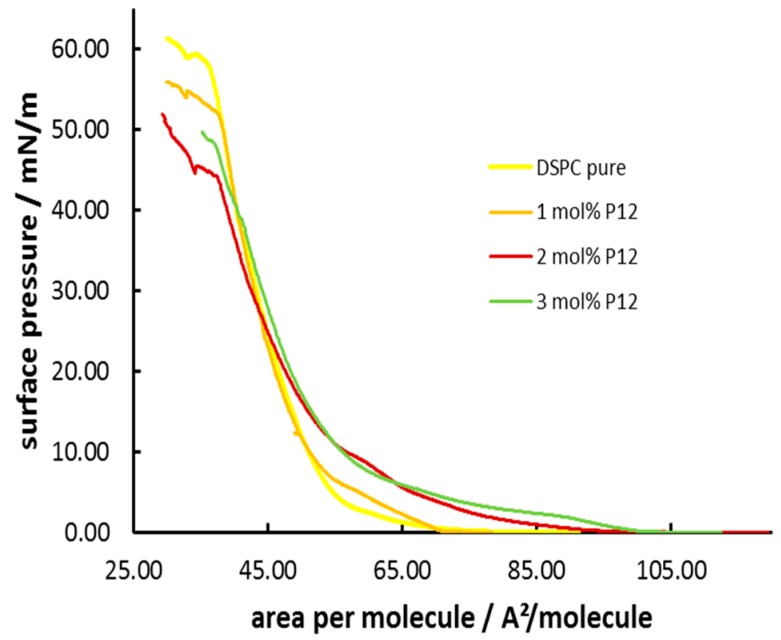
Isothermal surface pressure of a Langmuir-Blodgett monolayer at 25 °C with different amounts of lipopolypeptoids (pure DSPC, 1%, 2%, and 3% C_18_PSar_45_).

**Figure A2:**
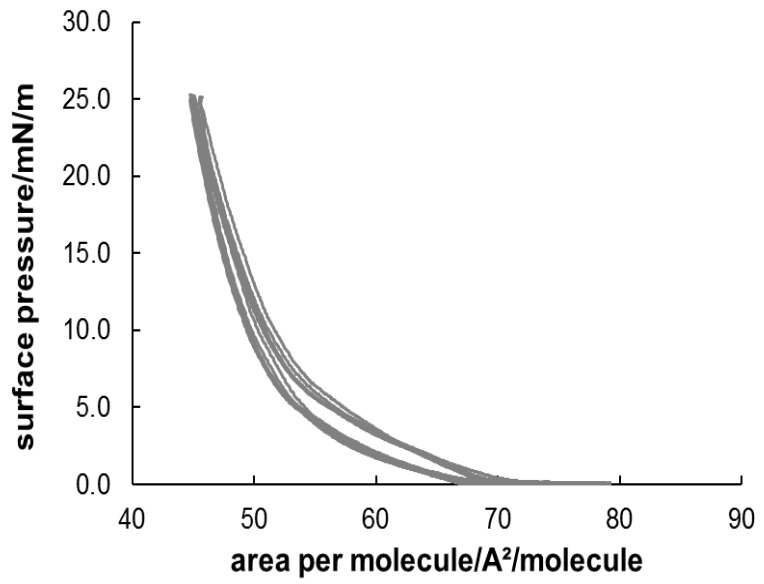
Isothermic compression and expansion hysteresis at 25 °C with 3% polymer content.

**Figure A3:**
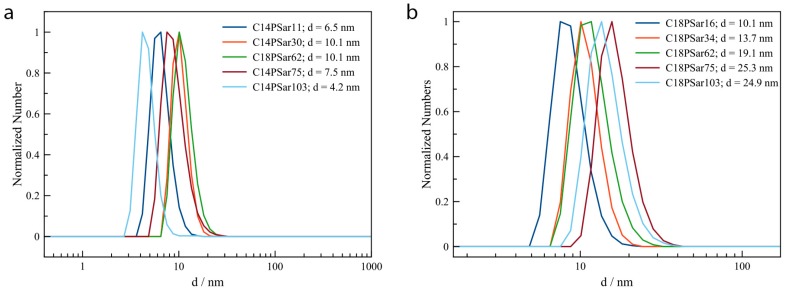
Number weighted diameter distribution of lipopolypeptoid determined by dynamic light scattering (DLS). (**a**) Series of tetradecyl amine (C_14_) based lipopolypeptoids; and (**b**) Series of stearyl amine (C_18_) based lipopolypeptoids.

The area per molecule when the membranes collapse remains constant at around 35 Å^2^/mol for 0%, 1%, 2%, and 3%. The surface pressure at the collapsing point is between 45 and 55 mN/m and thus comparable to monolayers formed by DSPC alone (65 mN/m) (Figure 5). To prove that no polymer was squeezed out during multiple compression and expension processes, cyclic measurements were carried out up to a surface pressure of 25 mN/m. This pressure was chosen to be well below the corruption point, but being in the solid-analogous phase. The hysteresis plots overlay with each other, indicating that none or only a minor loss of polymer occurs even for the highest lipopolypeptoid content of 3 mol% (Figure A2). 

## 4. Discussion

In contrast to the early work on lipopolypeptoids published by Gallot and coworkers [28,33] lipopolypeptoids displayed narrow molecular weight distributions and low dispersity indices of approximately 1.1 when synthesized according to the synthetic methods reported in this paper. According to MALDI-TOF MS data there are no detectable side products. Furthermore, we could demonstrate quantitative end group modification using anhydrides or isothiocyanates (ITC) by NMR studies (^1^H NMR and ^1^H-DOSY-NMR). Therefore, we do not see any reason for lipopolymer fractionation as reported by Gallot and coworkers [33]. Eventually the lack of control over the polymerization reported by the authors is related to the suboptimal choice of chloroform as a solvent for the reaction. As already demonstrated DMF [34,35,36], NMP or benzonitrile [18] should be preferred for the synthesis of such systems, since they ensure dissolution and enable the living ring opening polymerization of Sar-NCAs.

Kolliphor EL, formerly known as Cremophor EL, is a glycerol-based amphiphile, bearing about 35 PEG units in total and three hydrophobic tails. In comparison to the CMCs of Kolliphor EL of 90 mg/L lipopolypeptoids of comparable HLB values, e.g., C_18_PSar_11_, have a three-fold lower CMC of 27 mg/L [37]. In comparison to other PEGylated castor oils (e.g., PEG44 CO CMC: 958.2 mg/mL [38]) polysarcosine based lipopolypeptoids have an even ten-fold lower CMC at comparable HLB values. Since the A_2_ parameters in water between PEG and PSar are practically identical (unpublished data), this is somehow an unexpected result. But as PSar has an amide in each monomer unit, it has a less flexible backbone compared to PEG. This leads to a stretching of the polymer resulting in a closer packing and, therefore, to a higher micelle stability. Furthermore, the hydrophobic tails differ, so that the stacking of the hydrophobic domains also deviates.

These findings relate to the performed DLS measurements. Diameters found for the micelles are in the range of 9–25 nm. Polymers with a higher HLB value and a higher amount of polysarcosine assemble into larger aggregates compared to those with a smaller degree of polymerization. These studies show high dispersities on a micellar level ranging from 0.2–0.4. For C_14_PSar_103_ (*D* = 0.7) unimers and larger aggregates are detected in addition to the micellar fraction. Moreover, intensity weighted DLS displays larger structures of several hundred nanometers for some lipopolypeptoids (C_18_PSar_12_, C_18_PSar_30_, C_14_PSar_34_, and C_14_PSar_75_). However, these larger aggregates do not appear in the number weighted plot. This underlines that only a very small ratio assembles into large aggregates, being overestimated by intensity-weighted DLS. In comparison with PSar-based block copolymers, the lipopolypeptoid-based micelles are less uniform than those based on polypept(o)ides, [39] while they are comparable with aggregates formed by amphiphilic block copolypeptoids [40]. Likely, dispersities of assemblies can be lowered by methods for more controlled solution self-assembly, extrusion, or other preparation techniques. A formulation of these lipopolypeptoids with, for example, a lipid or hydrophobic molecules will lead to a better hydrophobic stabilization and, therefore, to more uniform structures. These studies, also of great interest, are beyond the scope of the current article.

The reported amphiphiles with a low degree of polymerization, show toxicity at 500 μM, which corresponds to 1.2 mg/mL. At 50 μmol no toxicity was detected in HeLa cells. Other polysarcosine-based materials have been reported to be non-toxic in HeLa cells up to 3 mg/mL by Birke et al. [34]. A polymeric hydrophobic block stabilizes aggregates more than an alkyl tail. The slower dynamics in these micelles compared to the reported lipopolypeptoids lead to reduced interference with cell membranes. Cremophor EL, as an example for a PEG-based system, shows toxicity at low concentrations (0.1 g/L) in endothelial cells [41]. 

Langmuir monolayers of pure DSPC have a phase transition to the solid analogous phase at 55 Å^2^/mol. This is the same value as reported by Hao et al. [42] It can also be seen, that with increasing PSar content the isotherms of the lipopolymers are shifted to higher area/molecule and a pseudo-plateau is reached at a surface pressure between 8 and 11 mN/m. PEG-based systems reported by Tanwir also showed pseudo-plateaus with an onset of 8 and 9 mN/m for 1% and 3% PEGylated lipid (degree of polymerization *X*_n_ = 45) [43]. The reported collapsing pressure of 59 mN/m is a little higher than for our systems (45–55 mN/m). Since the degrees of polymerization are comparable, this finding may be attributed to the differences in lipid parts of the lipopolymer. In comparison to the PEGylated DPPE-based system reported by Tanwir the reported lipopolypeptoids bear only a single alkyl chain. 

## 5. Conclusions

In this work, we report synthetic pathways to lipopolypeptoids with adjustable HLB values, precise control over molecular weights, dispersity indices, and end group integrity. The synthesized lipopolymers are of amphiphilic nature and self-assemble into micelles or more complex aggregates above their CMC in aqueous solution. The lipopolymers are non-toxic to cells up to a concentration of 50 μmol, which is a more than 10 times higher value comaqred to Cremophor EL. This finding points to a potential application as excipients in drug formulations. In addition, C_18_PSar_45_-based lipopolymers can be incorporated into Langmuir Blodgett monolayers based on DSPC up to a concentration of 3 mol % without altering its properties, which indicates the use of such lipopolypeptoids in the preparation of stealth-like liposomes. Therefore, the reported experiments are a first indication that polysarcosinylated lipids, named lipopolypeptoids, may be applied as bio-based excipients in drug or lipid formulations.

## Figures and Tables

**Table 1:** Polymer analysis of amphiphilic PSars.

Polymer	*X*_n_ (Calculated)	*X*_n_ (NMR)	*X*_n_ (MALDI)	*M*_n_ (GPC)	*Đ*
C_18_PSar_12_	10	12		5232	1.06
C_18_PSar_30_	30	30		7619	1.06
C_18_PSar_45_	50	45	47	13,933	1.07
C_18_PSar_64_	70	64		15,090	1.08
C_18_PSar_117_	100	117		24,890	1.13
C_14_PSar_11_	10	11		3960	1.05
C_14_PSar_34_	30	34		9035	1.07
C_14_PSar_53_	50	53	49	14,718	1.08
C_14_PSar_75_	70	75		18,220	1.12
C_14_PSar_103_	100	103		23,210	1.11

**Table 2:** Calculated HLB values and measured CMC.

Polymer	HLB value ^1^	CMC^2^/mg·L^−1^	Diameter of main peak (DLS/nm)	Distribution
C_18_PSar_12_	16.0	27	10.1	bimodal
C_18_PSar_30_	17.8	62	13.7	bimodal
C_18_PSar_45_	18.4	94	19.1	monomodal
C_18_PSar_65_	19.0	181	25.2	monomodal
C_18_PSar_117_	19.5	1181	24.9	monomodal
C_14_PSar_11_	15.7	213	9.1	monomodal
C_14_PSar_34_	18.4	-	12.7	bimodal
C_14_PSar_53_	18.9	-	16.4	monomodal
C_14_PSar_75_	19.1	-	19.2	bimodal
C_14_PSar_103_	19.4	-	24.4	trimodal

^1^ calculated according to W.C. Griffin: HLB = 20 × (1 − $\frac{M_{lipohilic}}{M_{lipopeptoid}}$); ^2^ measured using ring tensiometry (DCATIIEC) at 25 °C.
